# Association of Testosterone Levels and Serum Iron in Patients With Liver Abscess at a Tertiary Care Center

**DOI:** 10.7759/cureus.88477

**Published:** 2025-07-21

**Authors:** Kanchan Baitha, Nadeem Ahmad, Rishika, Yoshita Varma

**Affiliations:** 1 Department of General Surgery, Indira Gandhi Institute of Medical Sciences, Patna, IND

**Keywords:** ala, amoebic liver abscess, entamoeba histolytica, serum iron levels, serum testosterone

## Abstract

Background

Bacteria or parasites, such as amoebic agents, are the primary causes of liver abscesses. The study was conducted to determine the relationship between serum iron profile and testosterone levels among participants who were diagnosed with amoebic liver abscess (ALA).

Materials and methods

This was a nonrandomized observational study. It was conducted from August 2023 to July 2024 at the Department of General Surgery, Indira Gandhi Institute of Medical Sciences (IGIMS), Patna, India. A total of 30 patients were included in the study.

Results

The mean age of all 30 patients was 45.6 ± 7.9 years. Levels of serum ferritin were 350.6 ± 47.9 ng/mL, total iron was 225.8 ± 62.9 mcg/dL, transferrin saturation was 62.1 ± 16.5%, total iron binding capacity was 612.5 ± 104.3 mcg/dL, and unfractionated iron binding capacity was 410.7 ± 97.5 mcg/dL among patients. Serum testosterone was found to be 1425.7 ± 283.4 ng/dL among patients. This study reported a strong positive correlation between serum testosterone and serum total iron levels among patients with a p-value of <0.001.

Conclusion

The study found that both serum testosterone and iron profile were elevated in patients with ALA. Further, more prospective studies are needed to support our results.

## Introduction

Bacteria or parasites, such as amoebic agents, are the primary causes of liver abscesses, or collections of purulent material in the liver parenchyma; fungi are rarely involved [1]. While pyogenic liver abscess (PLA) affects more persons in industrialized regions of the world, amoebic etiology accounts for the bulk of cases in underdeveloped nations [2]. The human protozoan parasite *Entamoeba histolytica*, the cause of amoebiasis, is thought to infect about 50 million people worldwide and induce amoebic liver abscess (ALA) [3]. It is thought to kill 100,000 people annually, making it the third most common parasite cause of death after schistosomiasis and malaria [4].

For many years, ALA has been a prevalent issue in public health. The problem's scope is tremendous, especially in tropical regions. According to epidemiological research, adult males who regularly use the locally made alcoholic beverage toddy have a high prevalence of ALA [5]. Since there is insufficient data to conclude that these drinks are minimally contaminated with amoeba, a number of theories have occasionally been proposed to explain the pathophysiology of ALA in this susceptible population [6]. These ALA patients had surprisingly high rates of active colon bleeding, which results in extremely significant morbidity and mortality, according to a recent Indian study [7,8]. 

Recently, it was established that *Entamoeba histolytica* is frequently responsible for these clinically recognized liver abscesses. It has been proposed that the male hormone testosterone and alcohol-induced hepatic iron deposits play a significant part in the pathophysiology of ALA [9]. In mouse models, testosterone level influences susceptibility to ALA, despite the paucity of human data [10]. The study was conducted to determine the relationship between serum iron profile and testosterone levels among participants who were diagnosed with ALA.

## Materials and methods

Study design

This was a nonrandomized observational study. It was conducted from August 2023 to July 2024 at the Department of General Surgery, Indira Gandhi Institute of Medical Sciences (IGIMS), Patna, India.

Study population

A total of 30 patients were included in the study. The convenience sampling technique was preferred. The inclusion criteria for enrollment of patients were patients with confirmed ALA by radiological and histopathological findings, between 30 and 80 years of age, who were local palm drinkers, and presenting with an acute abdomen in the emergency and OPD. The exclusion criteria of the study were patients who were not able to provide informed consent, patients with a history of psychoses, those with a history of malignancies, patients with chronic renal and hepatic insufficiency, and chronic pancreatitis.

Data collection

Patients admitted with acute abdomen in the emergency and OPD were clinically examined, investigated with both laboratory and radiological studies. Complete blood count, liver function test, serum lipase and amylase, X-ray chest and abdomen, USG abdomen, and contrast-enhanced computed tomography (CECT) abdomen were done for the diagnosis. Following the clinical remission of symptoms, all patients were discharged with blood samples in a nonfasting state for testosterone and iron levels. Patients with a diagnosed hepatic abscess had their basic demographic information collected. For the purpose of analyzing total testosterone levels and a complete iron profile, which includes serum ferritin, total iron, transferrin saturation, total iron binding capacity, and unfractionated iron binding capacity, 3 mL of blood was drawn from each patient. To measure these characteristics, an enhanced chemiluminescence immunoassay was employed.

Statistical analysis

The estimation of sample size was done using the standard formula, i.e., N = 2(Zα + Z1-β)2 × σ²/Δ², where n is required sample size, Zα is equal to Z-value for type I error (α = 0.05), taken as 1.649, Z1 is Z-value for power (80%), taken as 0.8416, σ is standard deviation, and Δ represents minimum expected difference (effect size), assumed to be 2.

IBM SPSS Statistics for Windows, Version 25 (Released 2017; IBM Corp., Armonk, New York, United States) was used for analysis after the gathered data was imported into MS Excel (Microsoft Corporation, Redmond, Washington, United States). The data was presented as either n (%) or mean and standard deviation. Spearman’s rank correlation test was used to compare the quantitative values. P-values of less than 0.05 were considered statistically significant.

Ethical clearance

Under letter number 1075/IEC/IGIMS/2023 dated July 22, 2023, the Institutional Ethics Committee (IEC), IGIMS, Patna, Bihar, India, has acquired ethical approval.

## Results

In our study, the mean age of all 30 patients was 45.6 ± 7.9 years. Various other clinical features, such as abdominal pain, fever, cough, respiratory distress, dysentery, vomiting, and a lump in the right upper quadrant (RUQ) were also reported. Table 1 below represents the demographics of participants with ALA.

**Table 1:** Patient's demographics RUQ: right upper quadrant Data were presented as either n (%) or mean ± SD

Characteristics	Value
Age (in years)	45.6 ± 7.9
Clinical features	
-Abdominal pain	30 (100%)
-Fever	12 (40%)
-Cough	06 (20%)
-Respiratory distress	07 (23.3%)
-Dysentery	01 (3.3%)
-Vomiting	02 (6.6%)
-Lump in RUQ	08 (26.6%)

Laboratory findings such as hemoglobin was 10.9 ± 1.4 g/dl, platelet was 492.7 ± 172.8 x 10^3^/µL, and total leukocyte count was 15768.3 ± 4512.8 among patients. Radiological findings that were found among participants included hepatomegaly in 17 (56.6%) patients, hepatosplenomegaly in two (6.6%), pleural effusion in nine (30%), and lung collapse was observed in five (16.6%) patients. Clinical findings among participants are elaborated in Table 2.

**Table 2:** Laboratory and radiological finding among participants ALP: alkaline phosphatase; AST: aspartate aminotransferase; ALT: alanine transaminase Data were presented as either n (%) or mean ± SD

Clinical findings	Value
Laboratory findings	
Hemoglobin (g/dl)	10.9 ± 1.4
Platelet (x10^3^/µL)	492.7 ± 172.8
Total leukocyte count (n)	15768.3 ± 4512.8
Serum bilirubin (mg/dl)	1.04 ± 0.49
ALP (IU/L)	289.7 ± 35.8
AST (IU/L)	49.4 ± 12.7
ALT (IU/L)	45.9 ± 8.7
Serum amylase (U/L)	79.2 ± 9.1
Serum lipase (U/L)	155.8 ± 11.9
Radiological findings	
Hepatomegaly	17 (56.6%)
Hepatosplenomegaly	02 (6.6%)
Pleural effusion	09 (30%)
Lung collapse	05 (16.6%)
Ascites	06 (20%)
Vascular involvement	02 (6.6%)

Serum testosterone was found to be 1425.7 ± 283.4 ng/dL among patients. The levels of serum ferritin were 350.6 ± 47.9 ng/mL, total iron was 225.8 ± 62.9 mcg/dL, transferrin saturation was 62.1 ± 16.5%, total iron binding capacity was 612.5 ± 104.3 mcg/dL, and the unfractionated iron binding capacity was 410.7 ± 97.5 mcg/dL among patients. Table 3 represents the laboratory profile of the participants.

**Table 3:** Laboratory profile of the participants Data were presented as mean ± SD

Parameters	Normal Values	Value
Serum testosterone (ng/dL)	300-1000	1425.7 ± 283.4
Serum ferritin (ng/mL)	30-300	350.6 ± 47.9
Serum total iron (mcg/dL)	60-170	225.8 ± 62.9
Transferrin saturation (%)	20-50	62.1 ± 16.5
Total iron-binding capacity (mcg/dL)	240-450	612.5 ± 104.3
Unfractionated iron-binding capacity (mcg/dL)	-	410.7 ± 97.5

This study reported a strong positive correlation between serum testosterone and serum total iron levels among patients with a p-value < 0.001. The correlation between serum iron levels and testosterone levels is depicted in Table 4.

**Table 4:** Correlation of serum iron levels with testosterone levels Data were presented as mean ± SD. Spearman’s rank correlation test was used to obtain the p-value. A p-value of less than 0.05 was considered significant

Variables	Spearman's ρ (rho) value	p-value
Serum testosterone (ng/dL) vs. serum total iron (mcg/dL)	0.78	<0.001

Table 5 depicts the levels of serum testosterone in patients of different age groups. Testosterone level was found to decrease with respect to age in patients with ALA.

**Table 5:** Levels of serum testosterone in different age groups of patients with ALA ALA: amoebic liver abscess; SD: standard deviation Data were presented as mean ± SD

Age groups (in years)	Serum testosterone levels (ng/dL)
21-30	1289.6 ± 312.5
31-40	1089.6 ± 118.2
41-50	987.2 ± 102.1
51-60	770.9 ± 171.2

The relationship between age and transferrin saturation percentages in ALA patients is shown in Table 6. A significant moderate positive correlation was observed between age versus transferrin saturation, with a p-value of less than 0.001.

**Table 6:** Correlation of transferrin saturation percentages with age in patients with ALA ALA: amoebic liver abscess; SD: standard deviation Data were presented as mean ± SD. Spearman’s rank correlation test was used to obtain the p-value. A p-value of less than 0.05 was considered significant

Variables	Spearman's ρ (rho) value	p-value
Age vs. transferrin saturation (%)	0.53	<0.001

## Discussion

The goal of the current investigation was to determine whether testosterone levels and serum iron profiles were related in patients who arrived at the relevant center with ALA. Several researchers have previously linked iron and testosterone to the pathophysiology of hepatic amoebiasis [9]. The mean age of all 30 patients was 45.6 ± 7.9 years in our study. This age group is more vulnerable to liver abscess illness, according to research by Farhana et al. and Singh et al., which supports our findings [11,12].

A study by Jha et al. in 2019 reported that variables such as leukocytosis, higher serum bilirubin levels, and alcohol consumption complicate the disease [4]. Our study also reported leukocytosis, anemia, higher serum bilirubin levels, and alcohol consumption among patients with ALA.

In our study, patients had elevated levels of serum testosterone and total iron. Similar to our study, earlier research also showed that patients with ALA had elevated serum ferritin levels [11]. Several pathogenic mechanisms are mentioned as potential causes of high serum ferritin levels in inflammation, and ferritin, an acute-phase reactant, is predicted to be elevated in *Entamoeba histolytica* infections [13].

Liver abscesses are extremely rare, although intestinal amoebiasis is frequently found. These variations have been attributed to many processes, such as (a) immunologic changes that induce testosterone to predispose people, (b) increased alcohol intake in men, and (c) iron overload in the liver brought on by the high-iron content of locally made alcoholic beverages [3,14]. The explanation is that testosterone inhibits NKT cells from secreting IFN-γ, making males more susceptible to ALA. The male hormone testosterone may be a host factor that promotes the formation of ALA, according to animal models like hamsters [15]. Prior research from certain regions of Sri Lanka and India has demonstrated that an important risk factor for the development of ALA is the drinking of locally produced alcohol, such as toddy, which is typically brewed in unsanitary settings [14,16,17].

Limitations

This research has several restrictions. First of all, the study was limited in its capacity to be applied to other contexts or people due to its single site and small sample size. Second, because only patients who presented to our facility and satisfied the inclusion requirements were included, the use of convenience sampling may induce selection bias. Also, the cofounders were not assessed in the associations.

## Conclusions

This single-center observational study found that the levels of serum testosterone and total iron were found to be statistically significant among patients, with a p-value of <0.001. It was also observed that the levels of both serum testosterone and iron profile were elevated in patients with ALA. Therefore, to further confirm these findings, prospective and multicenter studies are essential.
